# Mood Influences the Concordance of Subjective and Objective Measures of Sleep Duration in Older Adults

**DOI:** 10.3389/fnagi.2016.00181

**Published:** 2016-07-26

**Authors:** Marion Baillet, Charlotte Cosin, Pierre Schweitzer, Karine Pérès, Gwenaëlle Catheline, Joel Swendsen, Willy Mayo

**Affiliations:** ^1^Université de Bordeaux, INCIA, UMR 5287 – Equipe NeuroImagerie et Cognition HumaineBordeaux, France; ^2^CNRS, INCIA, UMR 5287 – Equipe NeuroImagerie et Cognition HumaineBordeaux, France; ^3^EPHE, Laboratoire Neurobiologie et Vie QuotidienneBordeaux, France; ^4^Université de Bordeaux, ISPED, Centre INSERM U1219 – Bordeaux Population Health Research CenterBordeaux, France; ^5^INSERM, ISPED, Centre INSERM U1219 – Bordeaux Population Heath Research CenterBordeaux, France

**Keywords:** aging, actigraphy, ecological momentary assessment, sleep duration, mood

## Abstract

**Objective/Background:** Sleep plays a central role in maintaining health and cognition. In most epidemiologic studies, sleep is evaluated by self-report questionnaires but several reports suggest that these evaluations might be less accurate than objective measures such as polysomnography or actigraphy. Determinants of the discrepancy between objective and subjective measures remain to be investigated. The aim of this pilot-study was to examine the role of mood states in determining the discrepancy observed between objective and subjective measures of sleep duration in older adults.

**Patients/Methods:** Objective sleep quantity and quality were recorded by actigraphy in a sample of 45 elderly subjects over at least three consecutive nights. Subjective sleep duration and supplementary data, such as mood status and memory, were evaluated using ecological momentary assessment (EMA).

**Results:** A significant discrepancy was observed between EMA and actigraphic measures of sleep duration (*p* < 0.001). The magnitude of this difference was explained by the patient’s mood status (*p* = 0.020). No association was found between the magnitude of this discrepancy and age, sex, sleep quality or memory performance.

**Conclusion:** The discrepancy classically observed between objective and subjective measures of sleep duration can be explained by mood status at the time of awakening. These results have potential implications for epidemiologic and clinical studies examining sleep as a risk factor for morbidity or mortality.

## Introduction

Sleep has been proposed to serve as the biological “housekeeper,” helping to restore and repair the brain ranging from metabolite clearance (Xie et al., 2013) to cognitive functioning (Bonnet, 2011). Across normal aging, changes of sleep patterns and particularly sleep duration, have been extensively studied in the literature (Ohayon et al., 2004). Recent epidemiologic findings suggest that sleep duration plays an important role in cognitive functions and influences the risk of dementia mortality in older adults (Benito-León et al., 2014; Lo et al., 2016). Beyond cognition, sleep duration has important effects on a variety of medical conditions, including obesity, diabetes and hypertension (Knutson, 2012). For these reasons, the inclusion of sleep measures is increasingly used in elderly populations.

From a methodological perspective, polysomnography (PSG) is considered as the gold standard for assessing sleep parameters including electrical activity of the brain, eye movements, muscle contraction, and heartbeat (Sadeh et al., 1995). Despite its use in clinical studies, however, PSG may be difficult to apply in large epidemiologic or cohort-based investigations due to the possible constraint of requiring one or two nights of hospitalization (Camargos et al., 2013). Actigraphy is an alternative method of sleep assessment that overcomes several of these constraints associated with PSG (Sadeh, 2011). It involves the use of a wristband with accelerometers that detect and record movements with high sensitivity and it can be used in the patient’s home environment while permitting the sleep/wake cycle to be recorded over long time periods. Nonetheless, its use may also be costly when applied in large cohorts.

Faced with these methodological and economic issues, most investigations of sleep have used subjective self-report questionnaires (Ellis et al., 1981; Buysse et al., 1989; Johns, 1991; Netzer et al., 1999), presenting several advantages compared to objective techniques (e.g., inexpensive, brief and easy to use in large samples). They assess the patient’s own estimates of sleep duration as well as other factors that may impact sleep duration such as comorbid conditions and medications. Despite their importance in clinical and epidemiologic research, however, these instruments are more heavily influenced by self-perception and retrospective memory biases, in particular in older adults. Numerous studies have illustrated the relative inaccuracy of these evaluations compared to objective measures in middle age and older individuals and have suggested that this discrepancy increased with male gender, poor cognitive functioning, and behavioral disability (Regestein et al., 2004; Silva et al., 2007; Lauderdale et al., 2008; Van Den Berg et al., 2008; Kline et al., 2010; Cespedes et al., 2016).

Although extensive comparisons have documented the discrepancy between objective and subjective sleep measures, the identification of their underlying causes remain to be investigated. Existing evidence in the literature suggest that sleep and mood states are closely linked and that this relationship is complex and bidirectional (Kahn et al., 2013). The aim of this pilot-study is to examine the role of mood states in determining the discrepancy observed between objective and subjective measures of sleep duration in older adults. Considering the rapid daily fluctuations of mood (Golder and Macy, 2011), we assessed mood each morning over 7 days and collected both objective and subjective estimations of sleep duration over this same period.

## Materials and Methods

### Participants

**FIGURE 1:**
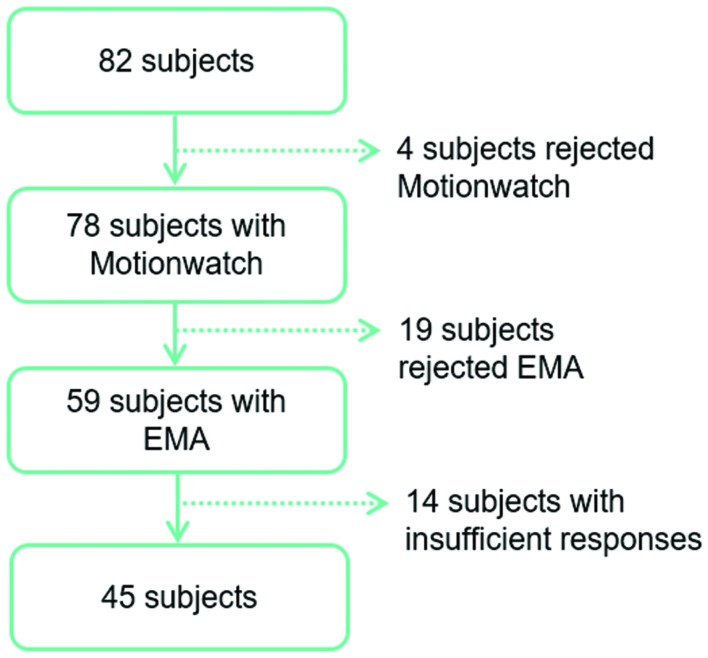
**Flow chart of participant selection**.

This study is a part of the AMImage2 research program started in 2012 and has included 200 participants as an ancillary study of the AMI cohort, an epidemiologic prospective study of health and aging. A more detailed description of the AMI cohort is provided elsewhere (Pérès et al., 2012). Study procedures were approved by the regional human research review board and all participants provided written informed consent. A subsample of participants was invited in the current study. After exclusion of failed recordings due to technical problems, participants were included if they were not using sleep medications, had no sleep complaints that had led to a diagnosis of sleep disorders and did not demonstrate significant depressive symptomatology (CESD < 16). From the 82 participants who meet these criteria, a total of 45 participants were included in the present study (**Figure 1**).

### Sleep Assessment

#### Objective Measures

Sleep parameters were recorded by a MotionWatch 8^®^ actigraph (Cambridge Neurotechnology, Cambridge, UK), equipped with a tri-axial accelerometer. The actigraph was placed on the non-dominant wrist during a period of 7 days and 8 nights. Participants were asked to press the event marker button in the center of the watch, when they started trying to sleep and when they woke up every day. Each participant could also complete a sleep diary indicating bedtime and waketime in order to facilitate interpretation of recordings. Activity was measured in 60 s epoch and the sensitivity threshold was set to the value of 40 counts (Cosin et al., 2015). Data were analyzed with MotionWare^®^, version 1.0.3.

Actigraphy allowed for the objective estimate of Total Sleep Time (TST), defined as the period between the onset and the offset of sleep. Sleep Efficiency (SE) was also calculated as the time spent asleep divided by the time in bed (given that influence sleep perception).

#### Subjective Measures

We used ecological momentary assessment (EMA), consisting of a structured electronic diary technique using a Samsung Galaxy S assessing current context, psychological phenomena and their interactions in daily life (Allard et al., 2014). Participants used EMA five times per day over a 1-week period. EMA assessments started the day following the hospital visit, and for each day, starting and ending hours of assessment were adapted to the individual’s typical daily schedule in order to limit sleep inertia even if this phenomenon cannot be totally excluded (Jewett et al., 1999). EMA was previously used and validated in community-dwelling elderly individuals (Bouisson and Swendsen, 2003; Allard et al., 2014). On average, each interview required less than 5 min to complete, and responses were saved in a time-stamped database.

For this study, we evaluated self-reported TST based on the question “How many hours did you sleep last night?” presented at the first assessment each morning. Participants were able to answer using a scale ranging from 0 h (no sleep at all) to 10 h or more. At least 3 nights of complete objective and subjective evaluations were required to include the participant’s data in the analyses. The great majority of EMA observations were within the hours proposed, with only 1,15% endorsing “10 h or more” of sleep time.

### Behavioral Assessment

#### Mood

Depressive symptoms were assessed using the Center for Epidemiologic Studies Depression scale (CESD) for the French population (Radloff, 1977). This scale consists of a self-report questionnaire containing 20 items which evaluate the frequency of symptoms or behaviors associated with depression. A score greater than 16 is suggestive of clinically significant depression and was applied as an exclusion criterion. In order to collect information about the inter-daily fluctuations of participant’s mood, we used EMA through the question: “To what degree do you feel happy now?” with responses recorded on a Likert scale of 1 (not at all) to 7 (extremely). This evaluation followed the self-reported TST questionnaire each morning at the first assessment.

#### Cognition

Global cognitive functions were controlled using the Mini Mental State Examination (Folstein et al., 1975). It includes 30 questions evaluating different cognitive dimensions such as time and space orientation, working memory, learning, attention, and language. A score inferior to 24 indicates poor cognitive functioning (Oosterman et al., 2008). As it might influence sleep perception, we assessed inter-daily fluctuations of episodic memory through EMA. This test consisted of the presentation of a list of 10 words followed by an immediate recall task and it occurred randomly over the course of the day.

### Statistical Analysis

Simultaneous utilization of actigraphy and EMA allowed us to obtain several measurement points per participant. Each night was then associated with an objective TST, SE, subjective TST as well as mood and episodic memory evaluations measured the following day. The agreement between subjective and objective TST was explored using a paired *t*-test. Considering that most of participants in the current study under and overestimated their TST over the week, an absolute difference between subjective and objective TST was calculated in order to obtain the magnitude of the discrepancy for each pair of observations.

To study the relationship between the magnitude of the discrepancy and SE, mood state and memory level, we used a multiple linear regression analysis using the magnitude of the discrepancy as outcome variable. We investigated possible confounds in our analyses; in that mood state assessed by EMA can be influenced by sleep duration and quality evaluated by actigraphy and memory level. For this, linear regression analysis was replicated by defining mood state as outcome variable. All analyses were performed with the IBM SPSS Statistics v.20 software (IBM Corporation, Armonk, NY, USA).

Finally, we used hierarchical linear modeling which is analogous to standard regression analyses but adjusted for dependencies among observations generated by each individual. The γ coefficients from these models represent the pooled within-person association between the predictor and the occurring outcome, *t*-ratios are the test statistic values for the null hypotheses that corresponding parameters are equal to zero. The outcome variable was defined as the magnitude of the discrepancy and age, gender, SE, mood state, and memory level as predictors. We also replicated our model by defining mood state as outcome variable. These analyses were conducted using HLM, version 6.03 (Scientific Software International, Inc., Skokie, IL, USA).

## Results

A flow-chart of participant selection is provided in **Figure 1**. From the initial cohort of 82 subjects, 45 participants (19 women and 26 men, mean age 75) were studied. Together, participants included in the study contributed to 175 valid nights for evaluation.

Subjects who visited the research center but refused to participate in the study were significantly similar in age (*p* = 0.180, *t*-test), sex (*p* = 0.892, Chi square test), MMSE (*p* = 0.275, *t*-test) and CESD (*p* = 0.569, *t*-test). Demographic, sleep and behavioral data of the studied sample and the comparison between objective and subjective evaluations are presented in **Table 1**. On average, the magnitude of the difference between objective and subjective TST was 1 h and 29 min (*p* < 0.001).

**Table 1:** Demographic, sleep and behavioral data.

Variables	Mean	±SEM	Minimum	Maximum
Demographic data				
^1^Age	75.39	±0.62	64	88
^1^Gender (female)	42 %			
Sleep data				
^1^Underestimation (%subjects)	62 %			
^1^Overestimation (%subjects)	02 %			
^1^Mixed (%subjects)	36 %			
^2^Actigraphic total sleep time	08:09^∗∗∗^	±00:05	04:39	10:55
^2^Self-report total sleep time	06:40^∗∗∗^	±00:06	02:00	10:00
^2^Sleep Efficiency	87.25	±00.60	52.8	97.5
Behavioral data				
^1^CESD	03.4	±0.52	0	12
^2^Happiness	05.5	±0.08	1	7
^1^MMSE	27.7	±0.25	24	30
^2^Episodic memory	05.0	±0.13	1	10

*^∗∗∗^p < 0.001 paired t-test;*^1^*n = 45 subjects;*^2^*n = 175 nights; SEM, standard error of the mean.*

Multiple regression analysis indicated that this magnitude was negatively associated with positive mood (β = –0.281; 95% CI:-0.509, –0.161; *p* < 0.001) but was not significantly associated with SE (β = –0.004; 95% CI:-0.024, 0.023; *p* = 0.962) or memory performance (β = 0.005; 95% CI:-0.105, 0.113; *p* = 0.947). In the second model, no association was found between mood states and actigraphic TST (β = 0.032; 95% CI:-0.116, 0.176; *p* = 0.687), SE (β = –0.085; 95% CI:-0.032, 0.009; *p* = 0.280) or memory level (β = –0.057; 95% CI:-0.130, 0.059; *p* = 0.454).

In order to account for within-subject dependence of observations, we also used multilevel modeling (**Table 2**). We observed a significant association between the magnitude of the discrepancy and mood states in that the more negative the state of mood was, the greater the discrepancy between the subjective and objective sleep durations was (*p* = 0.020). No association was found with the magnitude of the discrepancy and age (*p* = 0.247), gender (*p* = 0.100), SE (*p* = 0.883) and memory performance (*p* = 0.438).

**Table 2:** Demographic, sleep quality, mood, and cognition as predictor of the magnitude of the discrepancy.

Assessment variable	γ coefficient	SE	df	*t* ratio
Magnitude of the discrepancy				
Age	0.052	0.044	42	1.174
Gender	0.487	0.288	42	1.689
Sleep efficiency	–0.003	0.019	44	–0.148
Happiness	–0.271	0.112	44	–2.420^∗^
Episodic memory	0.050	0.064	44	0.782

*p < 0.05.

This within-person association of the magnitude of the discrepancy and mood was not affected by age (γ = –0.015, *SE* = 0.021; *t* ratio = –0.742; *p* = 0.462) or gender (γ = –0.040, *SE* = 0.243; *t* ratio = –0.165; *p* = 0.870). Moreover, we did not find an association between mood state and age (γ = –0.023, *SE* = 0.043; *t* ratio = –0.532; *p* = 0.597), gender (γ = –0.327, *SE* = 0.301; *t* ratio = –1.084; *p* = 0.284), actigraphic TST (γ = 0.081, *SE* = 0.071; *t* ratio = 1.141; *p* = 0.260), SE (γ = –0.006, *SE* = 0.010; *t* ratio = –0.611; *p* = 0.544), or memory level (γ = –0.007, *SE* = 0.029; *t* ratio = –0.244; *p* = 0.809).

## Discussion

The principal aim of this pilot study was to identify determinants of the discrepancy between objective and subjective measures of TST in older adults. In our sample of 45 community-dwelling elderly persons, we observed a significant discrepancy between sleep quantity evaluated by actigraphy and EMA. We found that 61% of participants’ nights had a magnitude of discrepancy superior to 1 h in estimation of sleep when compared to the actigraphy-based objective measure of TST. Importantly, the magnitude of this discrepancy was negatively associated with the subjective degree of positive mood at the time of awakening. There was no association between the magnitude of this discrepancy with age, gender, SE, or memory performance.

The current findings concur with previous reports indicating a discrepancy between objective and subjective measures of TST (Regestein et al., 2004; Silva et al., 2007; Lauderdale et al., 2008; Van Den Berg et al., 2008; Kline et al., 2010). As suggested by the literature, we did not use Pearson correlations to describe the concordance between subjective and objective measures because such coefficients assess only the linear relationship which may exist between two variables and not the extent of their agreement. All studies to date that have compared actigraphy and self-report measures have also averaged their results by individual, over a period of days. Such averaging of daily life variables ignores the fluctuations existing within individuals (Swendsen et al., 2000, 2011) and therefore provides less accurate indications of day-to-day phenomena.

Based on analyses of both between and within-person variance, several variables suspected to explain sleep misestimation were examined. Concerning the possibility that sleep duration estimates can be mediated by global cognitive functioning (Van Den Berg et al., 2008), we evaluated episodic memory capacity in light of evidence that this cognitive function shows a linear life-long decline (Hedden and Gabrieli, 2004). However, by assessing episodic memory performances each day through EMA, we observed that short term memory was not significantly implicated as an explanation for the difference between subjective and objective measures of sleep in older adults. This suggests that sleep duration misestimation may not be related simply to an inability to remember the number of hours slept. Sleep quality might represent another possible factor of the misestimation of sleep duration (Van Den Berg et al., 2008). In the current study we evaluated SE, an actigraphic indicator of sleep quality, and we did not observe any association between sleep misestimation and SE. It is important to note, however, that our sample was free of sleep complaints that had led to a diagnosis of sleep disorders, sleep medication and depressive symptoms. Considering the high comorbidity between these conditions with cognitive impairment, neuropsychological performances and SE, this result may be considered as consistent with previous findings (Dawson et al., 2008; Yaffe et al., 2011).

In the current study, we report an influence of mood states on the discrepancy between objective and subjective measures. This result is consistent with previous investigations suggesting associations between the Pittsburg Sleep Quality Index (PSQI), which is the most widely used self-report scale to assess subjective sleep parameters, and depressive symptoms, stress and anxiety (Grandner et al., 2006; Buysse et al., 2008). Although these studies showed a link between subjective sleep and mood, none of them evaluated the association of objective sleep duration and quality with mood. Our supplemental analyses showed that mood states were not influenced by sleep quantity or sleep quality evaluated by actigraphy the previous night. However, actigraphy cannot give insights into sleep architecture and it is possible that, for example, slow wave sleep adversely affects mood leading to the underestimation of total sleep duration (Bonnet, 1985; Finan et al., 2015). In addition, in our study mood was not associated with demographic variables and episodic memory. These results strengthen our primary hypothesis that a subjective feeling of insufficient sleep duration may be due to negative mood at the moment of sleep estimation. Considering that aging is associated with more positive overall emotional well-being and with greater emotional stability (Carstensen et al., 2011), any bias introduced by emotional instability is likely to have been small.

Actigraphy is being used increasingly in clinical and research studies as it has the advantage of providing objective information on sleep habits for several days consecutively in the patient’s natural sleep environment. Despite the fact that actigraphy has been validated in healthy subjects (Sadeh et al., 1995; Ancoli-Israel et al., 2003), it is not an exact sleep-wake indicator. It may slightly overestimate sleep in the case of participants resting quietly in their bed but who are actually awake, but this potential overestimation is probably limited to a matter of minutes (Sadeh and Acebo, 2002). Considering that the majority of our data reflects underestimation of sleep for a period longer than 1 h, bias introduced by the actigraphy device is likely to have been minor.

Concerning EMA, the feasibility of electronic ambulatory assessments has been previously demonstrated in healthy geriatric samples, as well as in elderly patients with neurological disorders (Cain et al., 2009; Johnson et al., 2009a,b). The benefits of EMA include the ability to provide momentary assessments of memory performance that may influence subjective evaluations, and the assessments provided in real-time reduce retrospective biases that may characterize other research methods. To our knowledge, the current study is the first to use computerized methods capable of confirming the temporal nature of associations between the magnitude of the discrepancy and its possible determinants in daily life.

Despite its increasing relevance to elderly populations, sleep remains a complex phenomenon to measure. Recent reviews suggest that health outcomes associated with sleep duration, in particular cognitive impairments, depend on how sleep duration is evaluated (Devore et al., 2016; Lauderdale et al., 2016). Considering the high prevalence of health outcomes associated with self-reported sleep duration, it may measure an important aspect of health that is not captured by objective techniques. It is important to note that this pilot-study utilized a sample of moderate size and we believe that further studies are needed to confirm our results in a larger cohort of elderly people. In addition, extending research of the discrepancy between both subjective and objective sleep evaluations throughout the lifespan would allow a better comprehension of this phenomenon. This research completes previous studies showing a discrepancy between both methods of sleep duration but also those reporting that perceived sleep quality is also different from what is objectively measured (Grandner et al., 2006; Buysse et al., 2008; Landry et al., 2015). These results could have important implications for epidemiologic studies examining sleep as a risk factor for morbidity and mortality.

## Author Contributions

MB, CC, WM, GC, and KP designed research. MB performed statistical analyses. PS and JS developed Ecological Momentary Assessment devices. MB, CC, WM, and JS wrote the paper.

## Conflict of Interest Statement

The authors declare that the research was conducted in the absence of any commercial or financial relationships that could be construed as a potential conflict of interest.
